# Supplementary material for: The Transcriptional Responses of Ectomycorrhizal Fungus, Cenococcum geophilum, to Drought Stress
Source: J Fungi (Basel). 2022 Dec 21;9(1):15. doi: 10.3390/jof9010015 (PMC9864566; doi:10.3390/jof9010015)
Supplement: Supplementary file 1 [file jof-09-00015-s001.zip › jof-2077976-supplementary.pdf]

Supplemental Table S1. Mycelia area of different isolates of *Cenococcum geophilum* under drought treatment.

Isolate no.	CK (cm^2)				10% PEG-6000(cm^2)					
	A	B	C	average	A	B	C	average	relative area	
ChCg001	7.64	7.83	7.58	7.68	2.57	2.42	2.42	2.47	32.1%	drought-sensitive
JaCg040	5.67	5.64	5.66	5.66	3.07	3.09	3.12	3.09	54.7%	
JaCg045	6.84	7.23	7.34	7.14	2.48	2.63	2.04	2.38	33.4%	
JaCg153	2.68	2.04	1.98	2.23	2.79	3.09	3.1	2.99	134.0%	drought-tolerant
JaCg205	3.11	3.27	3.2	3.19	3.12	2.92	2.84	2.96	92.7%	
JaCg037	3.81	3.72	3.71	3.75	3.69	3.57	3.99	3.75	100.1%	

Supplemental Table S2. Geographic and host information of different isolates of *Cenococcum geophilum* used in this paper.

**Isolate No.**	**Origin**	**Host tree species**
Chcg01	Yunnan Province, China	*Pinus yunnanensis*
Jacg37	Numazu Prefecture, Japan	*Pinus thunbergii*
Jacg40	Numazu Prefecture, Japan	*Pinus thunbergii*
Jacg45	Numazu Prefecture, Japan	*Pinus thunbergii*
Jacg153	British Columbia, Canada	*Picea glehnii*
Jacg205	Mount. Fuji (E35°20′21″, N138°47′22″,1554m), Japan	*Salix reinii*

Supplemental Table S3. Sequences of primers used for RT-qPCR analysis in this paper.

Primer	Sequence (5'-3')
K441DRAFT_702334-F	CGCAAGTCAGCGATCACCTA
K441DRAFT_702334-R	AGCGGGAGATGAGGGACTTA
K441DRAFT_186058-F	CGTGGTTTGGTCTTTCAGCG
K441DRAFT_186058-R	ACAACACCCTGCAAGTGTCA
K441DRAFT_660991-F	ACGGTGCTGTATCCAAGGTC
K441DRAFT_660991-R	TCCGAGGAAGACCACAAGGT
K441DRAFT_685815-F	AGTACCGCCATCGACTTTGG
K441DRAFT_685815-R	AGAATGGTAGCTGCGTCACC
K441DRAFT_554224-F	CCGTTTGATCCACTCCAGTT
K441DRAFT_554224-R	ATGAACACGGCTCTTCTCGT
K441DRAFT_571428-F	ATCCAACCGACCAACCTTCC
K441DRAFT_571428-R	CCGGGCTGTTGTGGTTAAGA
K441DRAFT_656193-F	TTGTACTGGGATGACCGTGC
K441DRAFT_656193-R	TACCATTTCGTCACGCCTCC
Cg-18S-F	CGGGTAACGGGGAATTAGGG
Cg-18S-R	CACCAGACTTGCCCTCCAAT

Supplemental Table S4. Sequencing quality and mapping rates of RNA-seq in different samples.

Samples	Clean reads	Clean bases	GC Content	%≥Q30
**C1CK1**	24,107,978	7,211,183,512	51.50%	95.31%
**C1CK2**	28,244,618	8,453,690,622	51.36%	95.05%
**C1P1**	29,036,299	8,690,372,210	51.29%	95.15%
**C1P2**	32,067,173	9,599,837,558	51.32%	95.13%
**J153CK1**	40,062,796	11,983,623,668	51.33%	95.48%
**J153CK2**	32,683,972	9,770,779,464	51.17%	95.22%
**J153P1**	26,341,572	7,879,053,798	51.08%	95.23%
**J153P2**	29,571,654	8,848,456,106	50.98%	95.12%
**J205CK1**	29,439,617	8,793,867,346	51.23%	94.99%
**J205CK2**	23,545,532	7,044,076,714	51.32%	95.33%
**J205P1**	29,725,557	8,896,335,498	51.36%	95.28%
**J205P2**	23,078,228	6,900,705,752	51.45%	94.93%
**J37CK1**	20,881,300	6,254,611,344	50.65%	95.35%
**J37CK2**	25,746,602	7,706,715,274	50.88%	95.24%
**J37P1**	22,017,422	6,578,725,336	50.76%	95.28%
**J37P2**	23,126,857	6,922,748,292	50.79%	95.18%
**J40CK1**	20,828,942	6,227,599,572	51.16%	95.43%
**J40CK2**	35,461,701	10,616,996,240	51.25%	95.38%
**J40P1**	22,544,867	6,752,388,660	51.44%	95.20%
**J40P2**	20,373,524	6,092,161,376	51.36%	95.28%
**J45CK1**	19,202,220	5,746,472,114	51.33%	95.23%
**J45CK2**	21,889,402	6,556,759,762	51.33%	95.12%
**J45P1**	28,853,247	8,638,831,930	51.22%	95.02%
**J45P2**	21,690,649	6,491,093,662	51.25%	95.18%
